# Spatial Distribution of Off-Host Stages of *Tunga penetrans* in the Soil within the Home Range of Nine Infected Dogs in An Endemic Tourist Area in Brazil

**DOI:** 10.3390/tropicalmed8020098

**Published:** 2023-02-02

**Authors:** Anderson Vieira de Jesus, Anaiá da Paixão Sevá, Paula Elisa Brandão Guedes, Katharine Costa dos Santos, Tatiani Vitor Harvey, Gabriela Mota Sena de Oliveira, Thammy Vieira Bitar, Fernando Ferreira, George Rêgo Albuquerque, Renata Santiago Alberto Carlos

**Affiliations:** 1 Departamento de Ciências Agrárias e Ambientais, Universidade Estadual de Santa Cruz (UESC), Ilhéus 45662-900, Brazil; 2 Department of Veterinary Integrative Biosciences, Texas A&M University, College Station, TX 77843, USA; 3 Departamento de Medicina Veterinária Preventiva e Saúde Animal, Faculdade de Medicina Veterinária e Zootecnia (FMVZ-USP), São Paulo 05508-270, Brazil

**Keywords:** dog home range, geoprocessing, neglected tropical disease, sand flea

## Abstract

Tungiasis is a neglected disease caused by the sand flea *Tunga penetrans*, and dogs are considered the main reservoirs in Brazil. This study aimed to identify the role of dogs as tungiasis dispersers and to investigate the presence of *T. penetrans* in the soil of an endemic tourist area. Nine dogs infected by *T. penetrans* were included in this study and received GPS collars to analyze their movement through the village. Duplicate sand samples were collected in different areas of the community. Those areas were classified as peridomicile (n = 110), open area (n = 110), beach (n = 50), and river (n = 58). The analysis of the points recorded by the collars showed that the dogs roamed throughout the community, potentially facilitating the spread of the disease. Samples contaminated with developmental forms of the parasite were found in the circulation area of three dogs. Adult fleas were found in 3/328 samples. These data emphasize that infected dogs’ roaming can influence the fleas’ dispersion in the soil. Statistically, none of the study variables had a significant correlation (*p* > 0.5) with the presence of *T. penetrans* in the area analyzed. This study was the first to assess infected dogs’ role as tungiasis propagators.

## 1. Introduction

*Tunga penetrans* is a hematophagous ectoparasite that affects mammals [1] and causes tungiasis, a zoonosis characterized by the penetration of the female flea into the host’s epidermis [2]. This disease occurs mainly in poor communities in developing countries, being reported in Latin America and sub-Saharan Africa [1,3,4]. In Brazil, its occurrence has been widely described in indigenous [5] and rural [6,7] communities, slums [8,9,10], and fishing villages [9,11]. The state of Bahia is located in the country’s Northeast region, and tungiasis was reported as endemic in a fishing community in the municipality of Ilhéus [4,12,13].

The domestic animals most associated with human infection are dogs, cats, and pigs, which become infested with the flea present in the environment, and in turn propagate their eggs in the environment [9,11]. After the parasite penetrates the host’s skin, it feeds on the host’s blood and lays eggs, which develop within three weeks and are released mature by the parasite into the environment. Then, the female flea’s involution and death occur, completing its life cycle and leaving a lesion on the host’s skin that will heal slowly [11,12].

*T. penetrans* larvae can be found in various soil types, but those most prone are dry and sandy [14]. In West Africa, studies have indicated that sand floors of houses were an essential indicator for the occurrence of tungiasis in the area; however, the inner floor commonly resembled the outer floor [15].

In Brazil, a survey that analyzed soil samples from homes in endemic regions located in the Northeast (state of Ceará), Midwest (Mato Grosso), and North (Roraima) regions, in dwellings with sand floors and organic material, showed that 28/134 (20.8%) of the samples were positive for *T. penetrans*. In favela (slum) residences, 9.3% of the samples were positive, while in malocas the prevalence was 32%, demonstrating that *T. penetrans* can complete its cycle in an indoor environment [10]. Additionally, Harvey et al. (2019) [13] proposed that the free access of dogs to the streets favors the maintenance of the parasite in the environment, and therefore of the infection. This last study cited was carried out in an economically vulnerable community visited by tourists in the state of Bahia. In this village, previous studies have shown that the infection is endemic and not seasonal, unlike other regions where there is variation of infection according to the season of the year. The village in question has sandy soil [12,13], which favors the development of the parasite in the environment [15].

Considering that tungiasis is a neglected disease and considering the role of dogs as one of the main disseminators of *T. penetrans* in Brazilian communities, environmental studies focused on soil contamination contribute both to a better understanding and to the control of the disease in animals and humans. To date, there are no published georeferenced data on canine tungiasis in the world. Thus, in order to better understand the role of dogs as parasite dispersers in an endemic community in the municipality of Ilhéus, Bahia, Brazil, we investigated the presence of *Tunga* spp. fleas in the soil, their developmental stages, and their association with the soil characteristics and the canine population, checking the living area of positive dogs.

## 2. Materials and Methods

### 2.1. Ethical Considerations

This study was approved by the Ethics Committee on Animal Use of Santa Cruz State University (CEUA-UESC) under number 013/2020.

### 2.2. Study Area

**Figure 1:**
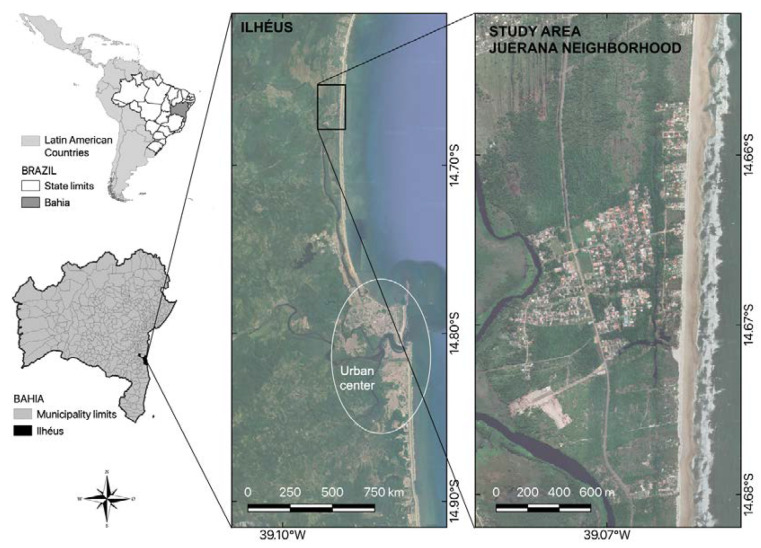
Study area in Vila Juerana on the northern coast of Ilhéus, Bahia, Brazil.

The study was carried out in a tourist community called Vila Juerana, located in the coastal area of the northern rural region of the municipality of Ilhéus, Brazil (Figure 1), between January and March 2021. Ilhéus’ predominant biome is the Atlantic Forest, and it has a humid tropical climate [16].

### 2.3. Selection of Dogs and Home Range Calculation

#### 2.3.1. Selection of Dogs

**Figure 2:**
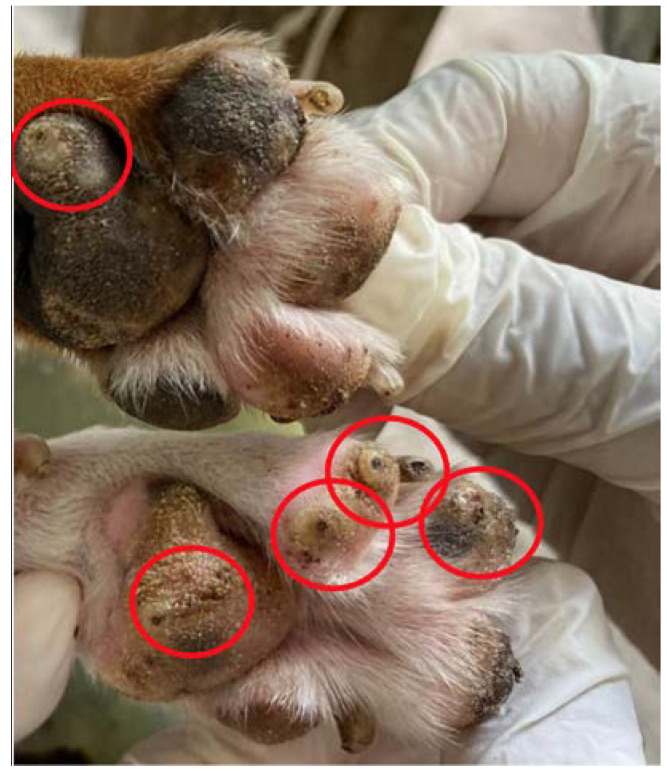
Pad and digits of a dog paw infected with *Tunga penetrans* in stage III (circles), according to the Fortaleza classification.

The following characteristics were used as selection criteria for the dogs: (a) the presence of active tungiasis lesions in stages I to III (Figure 2), according to the Fortaleza classification [17]; (b) age over one year old; (c) free access to the street, a key factor for the movement to be spontaneous, without human interference; and (d) weight greater than 10 Kg, to facilitate carrying the GPS collar. The infected animals were identified by physical examination during home visits, with mechanical restraint.

Of the nine dogs that participated in the study, seven were male and two were female, all uncastrated, aged between two and eight years (median = 5; standard deviation = 2.56), with weight varying between 10.8 and 25.6 kg (median = 15.0; standard deviation 4.7). The animals had owners, but had a semi-domiciled lifestyle, with free access to the entire community. All dogs had vital lesions compatible with tungiasis (Table 1). The dogs also showed acute clinical signs related to tungiasis, such as pruritus, hyperemia, mutilation and edema of the pads.

#### 2.3.2. Distribution of GPS Collars

Tigrinus^®^ brand collars were used, which weighed approximately 120 to 200 g each. Before placement on the animal, the collars were configured to record geographic coordinates in UTM every 10 min, with date and time. This interval was determined considering the robustness of the information and the occurrence of failures in capturing the coordinates, so that the battery lasted the entire period (seven days) of use without jeopardizing the recording of the animals’ movement.

The collars were covered with insulating tape to prevent water from damaging the electrical part of the device. For better fixation of the collars on the dogs, a commercial collar was used to adjust the GPS device to the neck of the animals. Finally, they were covered with waterproof aluminum foil tape to prevent them from falling or being removed by unauthorized persons. The dogs wore the collars for seven days.

#### 2.3.3. Necklace Data Collection

To access the collar data, the Tigrinus^®^ program, version 2.2, was used. All captured geographic coordinates of each animal were plotted on maps, along with the estimated average home range, to visualize the circulation during the selected period.

#### 2.3.4. Living Areas

The analysis of animal movement was used to understand the living-use area, since at larger scales, most animals have a tendency to remain in a defined region or home range. We used the model proposed by Calabrese, classified as a continuous-time stochastic process (CTSP) [18], in which we recorded geographic area traveled by the animal, and the time interval between the coordinates. The model considers three features: position autocorrelation (position observations that are closer together in time); velocity autocorrelation (velocities that are closer together in time and position); and home range (tendency to remain in a particular area). The analyses were performed with the R program (R Core Team, 2017), version 3.4.2, using continuous-time motion modeling packages (ctmm). First, we built a standard workflow that allowed visual diagnosis, followed by candidate model identification, and then maximum likelihood fitting and AIC-based model selection. Once an accurate CTSP was fitted and selected, we quantified the home range areas via autocorrelated kernel density estimation or estimation of occurrence distributions via time-series kriging. Finally, we estimated the 95% home range area, with 95% confidence intervals, to exclude possible biased records, rarely visited areas, or distant areas for a relatively short period [18].

The home range was generated in shapefile format, and was used to prepare maps and calculate the areas with the QGIS program.

#### 2.3.5. Comparison of Sand Collection Points and Dogs’ Home Range

The coordinates of soil sample collection (with data on presence or absence of parasites) and the dogs’ home range were plotted on the map, by using QGIS software (version 3.10), and overlap was identified.

### 2.4. Soil Analysis for the Presence of Tunga spp.

#### 2.4.1. Demarcation of Soil Collection Points

The soil collection points in the community were previously demarcated in space using a satellite image (from Google) in the QGIS program. Within the study area, four categories were considered with different soil characteristics and behavior of animals and humans: (1) peridomicile (<7 m from the houses), (2) open area (>7 m from the houses), (3) river (<8 m from the river bank), and (4) beach (close to the sand strip). Within the area of each category, the allocation of points was randomly defined. Since no reports of soil sampling were found for *Tunga* sp. in any studies, sampling was performed considering prevalence of 50% (maximum value to reach the lower final confidence interval of final prevalence), acceptable error of 5.5%, confidence level of 95%, and infinite population, resulting in a total required for the collection samples at 317 points [19].

A greater number of samples were obtained from the peridomicile and open areas since these areas had the highest frequency of hosts and were the same for both (110 in each). Fifty samples were obtained from the sand area of the beach and 58 along the river, totaling 328 samples. Even though the coordinates of the sampling areas had been previously determined on the map, the geographic coordinates were recorded again with the aid of a Garmin^®^ GPS map 78 configured for the UTM system.

At each collection point, the following environmental factors were recorded: (1) presence of organic waste (dry leaves, plant and food remains, chicken and other animal feces), (2) presence of animals during collection (chicken, dog, cat, duck, none), (3) presence of shade at some point during the day (morning, afternoon, both periods, absent; for example: river with shade throughout the day; clay soil and sandy beach with shadows only in some places; peridomicile with shade only in the morning and dirt floor; sunny area all day, with sandy soil; open area with shade throughout the day, with sandy soil), and (4) the type of soil.

#### 2.4.2. Collection of Soil Samples

The collection was carried out with a small garden spade, 270 × 56 × 40 mm. The surface layer of the soil, one centimeter deep [10], was collected with the spade passed horizontally over the soil. Samples from each point were collected in duplicate for better accommodation and non-compaction of the sand. The collected samples were placed individually in 250 mL disposable plastic cups, identified, sealed, and kept at ambient temperature for further analysis. Immediately after sample collection, the plastic cups were sealed with lids. Before opening the lids, the plastic cups were placed inside a plastic bag and the lids were opened, so that if there were any adult fleas they would leave the cup and stay inside the bag. Before starting the analysis of the sands, the plastic bag was carefully checked for live fleas. If there were, fleas were captured before removing the cup from the plastic bag. The collected samples were sieved to remove the largest particles such as stones. In the sequence, the sand was examined in small portions in the dissecting microscope. All samples were collected in the morning, between 6:00 a.m. and 11:00 a.m. [10], and analyzed in the afternoon.

#### 2.4.3. Investigation of the Presence of *Tunga* spp. in the Soil Samples

Soil samples were examined at the Laboratory of Veterinary Parasitology at the Veterinary Hospital of Santa Cruz State University (HV-UESC), up to three days after collection and once a week for three consecutive weeks. For this purpose, the samples were placed separately in 90 × 15 mm Petri dishes, the stages of *T. penetrans* were determined by placing them on slides fixed in Canada turpentine and observed with a stereoscopic magnifier with 2× magnification. When the stages were found, they were placed in a flask with alcohol to carry out the morphological evaluation and identification of the species and the development phase, with the aid of a binocular microscope at 40× magnification, as described by Monteiro (2007) [20].

### 2.5. Statistical Analysis

To compare the variables of type and characteristics of the sampling sites (presence of organic material, frequency of shadow, species of animals present, type of soil) with the presence of the parasite in the soil, Chi-square or Fisher’s exact tests were performed, considering *p* < 0.05 as significant, using the free software R (version 3.5).

## 3. Results

**Figure 3:**
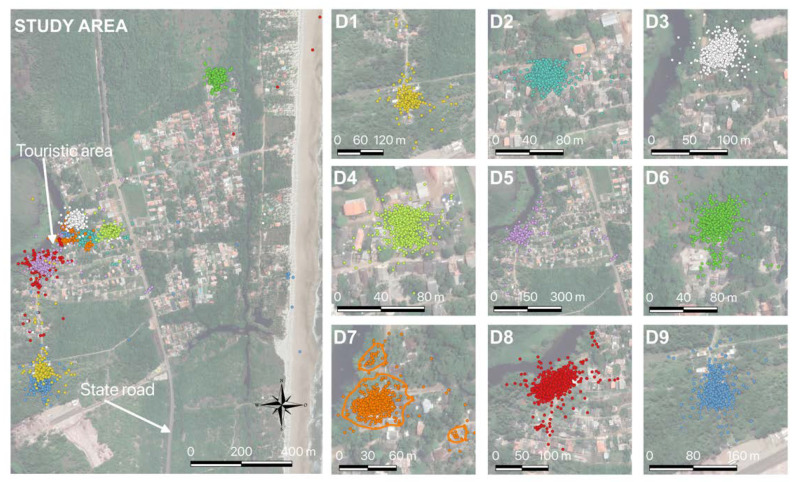
Co-ordinates of movement of dogs in Vila Juerana, Ilhéus, georeferenced by a collar with GPS. Each dog is represented by a different color and, for D7, there is also a polygon representing the home range. Legend: **D1**–**D9** are the identification of each dog.

All nine dogs had their routes recorded by GPS collars, seven males and two females (D4 and D6). Two dogs walked to the beach (D8 and D9), one of them (D8) traveling 1635 m and the other (D9) 960 m from their homes to that location. Another four animals remained closer to their homes (D1, D2, D3, and D4). Six dogs were from areas close to the river (D2, D3, D5, D7, and D8), and three of them crossed the riverbank (D1, D5, D8), close to the community’s tourist area (Figure 3).

The fact that these dogs visited the beach, crossed the river, or frequented distant and random spots does not mean these are spots that are part of the animals’ home range (Figure 3). According to the statistical program, they may have been visited rarely or only for short periods. Figure 3 shows the circulation points of animal D7 (as an example) and the representation of its home range use.

**Figure 4:**
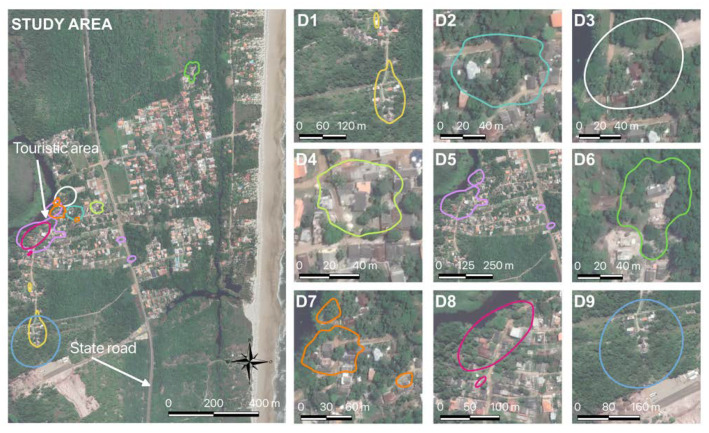
Home range of all dogs, including overlays (left map) and dogs alone. Legend: **D1**–**D9** are the identification of each dog.The average living area of the animals was 6502.0 m^2^ (95%CI: 5958.2–7066.6), with the smallest area being 2310.9 m^2^ (95%CI: 2148.3–2479.8; D4) and the maximum 38,807.7 m^2^ (95%CI: 32,574.1–45,553.8; D9) (Table 2).

The home ranges of the dogs varied in size, and some did not show continuity, with more than one area of use (D1, D5, D7, and D8). Only two dogs did not share their home range with another dog in the study, females D4 and D6 (Figure 4, left map), which also had one of the lowest dispersions.

**Figure 5:**
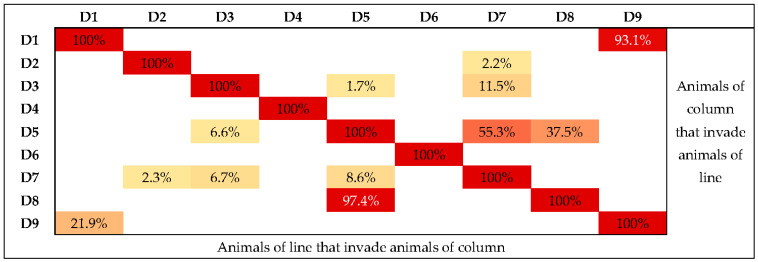
Percentage of shared living areas. Below the diagonal: Animals from the row that invaded the area of the animals in the column; and above the diagonal: animals in the column that invaded the area of animals in the row. Legend: The color grading represents the intensity of shared areas (the more intense the color, the greater the shared area).

Animals with more extensive home ranges shared areas with other animals. This was the case of dog D9, who shared 93.1% of the area of D1 (8498.2 m^2^), and dog D5, who visited 8.6% of the area of D7 (2109.6 m^2^) and 97.4% of that of D8 (9236.5 m^2^) (Figure 5). Notably, the owner of animals D1 and D9 was the same. Animals D2 and D7 had similar shared ranges between them (2.2% and 2.3%, respectively, 84.9 m^2^), as did animals D7 and D3 (11.5% and 6.7%, respectively, 437.4 m^2^). Animals D5 and D7 also shared their areas with a small percentage (1.7% and 6.6%, respectively, 427.8 m^2^). The dog that most shared territory with the other animals was D7. However, it had one of the smallest home ranges.

**Figure 6:**
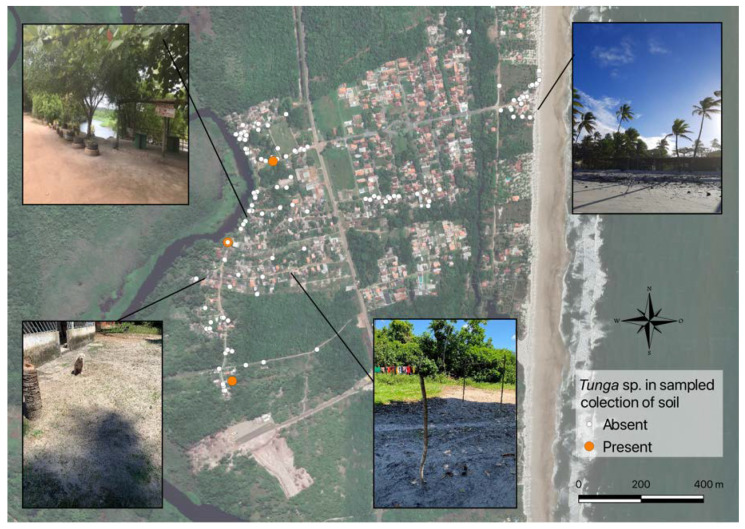
Areas where soil samples were collected with and without *Tunga penetrans*. Examples of environmental and soil characteristics can be found in the figures: Top left: river with shade throughout the day and clay soil and sandy beach with shadows only in some places; bottom left: peridomicile with shade only in the morning and dirt ground; upper right: sunny area all day, with sandy soil; and bottom right: open area with shade throughout the day, with sandy soil.

**Figure 7:**
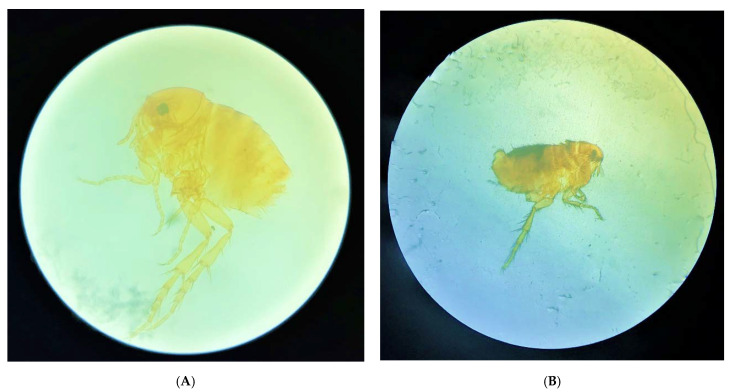
(**A**) Adult *T. penetrans* at 10× magnification under optical microscope. (**B**) *C. canis* at 4× magnification under optical microscope.

Of the 328 soil samples, only three (0.9%) were positive for adult fleas, the only stage of *T. penetrans* found in positive samples (Figure 6). The home range of the dogs with the highest proportion of positive samples was the peridomicile (Table 3). The first had nine *T. penetrans* fleas (Figure 7A) and one *Ctenocephalides canis* (Figure 7B), and the third sample contained three *C. canis* and one *T. penetrans* fleas. The two samples containing *T. penetrans* and *C. canis* came from the peridomicile, corresponding to 1.8% (2/110) of the total samples. The sample that only showed *T. penetrans* was from the open area, which corresponded to 0.9% (1/110) of the total samples and was collected in the middle of a street (occurrence further north). It is noteworthy that dogs D1, D9, D5, and D8 had *T. penetrans* confirmed in the sand within their home range. Images of the places where samples were collected with different types of shading and soil are shown in Figure 6.

The area occupied by animal D5 had the highest number of samples (15) of sand compared to the areas of the other animals, thus also having the highest percentage compared to the total samples. The prevalence of parasites in the area of D1 was 8.33% (CI 95%: 0–23.97); of D9, it was 50% (CI 95%: 0–100); of D5, it was 6.67% (CI 95%: 0–19.29); and of D8, it was 20.0% (CI 95%: 0–19.29) (Table 3).

For the variable organic matter, the presence of other animal species at the time of collection and shading in relation to the presence or absence of fleas, the tests (Chi-square) were not performed, due to the number of negative samples in the different categories. Of the 328 samples collected, 302 came from sandy soil and 26 from clay soil. All the positive samples were from sandy soil, but this prevalence was not statistically significant (Fisher’s exact test) (Table 4). However, with regard to the variable organic matter in the soil, all three positive samples had dry organic material (*p* = 1). Regarding the presence of animals, in the collection sites with *T. penetrans,* there were only dogs. The three samples with *T. penetrans* were found in places with shade in the morning and afternoon. These sites were close to trees that prevented direct sunlight on the ground due to their canopy. Finally, the type of soil that presented *T. penetrans* was sand, with three positive samples.

## 4. Discussion

Living with humans and the semi-restricted way of handling dogs make them one of the most significant challenges for controlling tungiasis in Brazil and the rest of Latin America [9,10]. Overlap of *Tunga* spp., observed mainly in semi-restricted animals, increases the risk of human and environmental infection [4,10,12,13]. In endemic areas such as Vila Juerana, this risk is exacerbated due to the wide home range of these animals, allowing dispersion of the parasite between households, as evidenced in this study.

Monitoring the movement of infected dogs allowed the identification of areas with the highest risk of environmental contamination in the community. The dogs had average living areas ranging from 2310.9 m^2^ to 38,870.7 m^2^. Some were restricted to their owners’ homes, while others moved to more distant places, as was the case of two dogs (D8 and D9) who wandered to the beach, and three that crossed the river (D1, D5, and D8). In this context, it is valid to infer that the tourists who visit the community have a risk of infection since they commonly go barefoot to the leisure areas that border the river and the beach, where the act of lying on the sand also favors ectopic infections. This risk is linked to flea eggs being constantly released into the environment from the lesions of parasitized dogs. Consequently, the greater the movement of these animals, the greater the area of contamination within the community, inside and outside the dwellings. We also point out that tourists commonly take their animals for walks in these places (personal observation), favoring the infection of these animals and the spread of sand fleas beyond the limits of the community.

We observed that in addition to places close to houses, the living areas of most collared dogs included the tourist area, with bars along the river. The bars attract dogs for their food and the river is used by tourists to bathe and by residents to wash clothes, bathe themselves, and bathe their dogs. Therefore, local residents and visitors often walk around barefoot and sit on the ground. All these factors are potential sources of human infection by *Tunga* spp., which can cause mild to severe injuries, with associated secondary infections [21,22]. To the best of our knowledge, no studies have been published evaluating the home range of semi-domiciled dogs associated with the dispersion of parasites that cause tropical diseases, so there is a need for further research similar to that reported here.

The analysis of the movement of the dogs showed that their home range was primarily concentrated in the peridomicile (close to their respective residences). This fact, added to the identification of fleas in peridomestic soil samples, albeit without statistical association, showed the relevance of contamination of peridomestic soil for the perpetuation of the parasite within the community [9,10], and indicates a higher risk of infection for families with dogs infected.

The GPS collar can only be used on medium and large dogs (>13 kg). This condition reduced the number of infected semi-restricted dogs participating in the study, since most dogs in the community were small-sized. The fact that the dogs sampled circulate over a wide geographic area suggests that the smaller infected dogs also circulate over large areas. We emphasize that we observed a high number of small semi-domiciled dogs in the community that were positive for tungiasis, which leads us to assume that parasite dispersion may be greater in this village.

Although 0.9% of the evaluated soil samples were positive for the presence of *T. penetrans*, we cannot state that adjacent areas to those sampled do not have any other form of *T. penetrans* development. However, since the area studied is endemic for the parasite, we believed all development forms would probably be present. It is noteworthy that the failure to find the parasite in the soil samples could have been due to the presence of detritus in the soil and the shine of the sand under the magnifying glass, which greatly hindered this evaluation.

Rainwater can carry the eggs and larval stages through the soil, since they are tiny and can reach deeper parts of the sand [10]. In the evaluated region, the frequency of rainfall is high at various times of the year, especially when the analyses were carried out. This fact also may have hindered the collection of parasitized samples since the maximum depth of collection was 1 cm, according to the method described by Linardi et al. (2010) [10]. The type of soil collected (mainly sand) may also have affected the frequency of parasites found, by allowing eggs and larvae to infiltrate into deeper layers. In this sense, the soil collected by Linardi et al. (2010) [10] was characterized by them as being of the laterite type, which likely made it difficult for *T. penetrans* life forms to penetrate deeper layers. These data suggest the need for studies in the region with more samples collected at different depths and seasons of the year for a better understanding of this issue. Thus, the failure to find a large number of positive samples does not mean there are no reproductive forms of *T. penetrans,* since this is an endemic region. It should also be noted that dogs often scrape the ground and make holes, facilitating their contact with fleas in layers deeper than 1 cm.

Additionally, two samples were also positive for *C. canis*, indicating the coexistence of the parasites in the same environment. In this respect, Linardi et al. (2010) [10], in their study of malocas and houses in slum areas of northeastern Brazil, also found them positive for *T. penetrans* and *C. felis*, indicating that more than one species of flea was present in the study area, with cycles coexisting in parallel without harming each other’s development or competing for space.

The mapping of collection points is vital for indicating possible sites of agent dispersion, thus helping to control the parasite in the environment and prevent humans and animals from becoming even more contaminated. Hence, there is a need to implement mapping of neglected tropical diseases to help public health authorities formulate effective policies to control the disease [23,24,25,26]. Because the community in this study is a tourist point, it receives people from other areas of Brazil (who often bring pets) and the world. In this context, other authors have described cases of tungiasis in tourists who visited endemic areas of tropical and subtropical countries [27,28]. Therefore, knowledge of the types of habitats where *T. penetrans* is present can help improve the safety of both local residents and tourists. As further preventive measures, we suggest research into medicinal products that interfere in the parasite’s life cycle and education of dog owners about avoiding the high frequency of dogs circulating without supervision, since canines are most often infected by the parasite.

## 5. Conclusions

Monitoring the movement of dogs infected by *Tunga* spp. is a robust tool to identify the dispersion of the parasite in the soil, by identifying priority areas for prevention and control by application of insecticides. Peridomicile areas favor the maintenance of animal reservoirs and should be prioritized in disease control campaigns. Environmental contamination due to the free movement of infected dogs can increase the risk of human infection inside and outside endemic tourist areas.

## Figures and Tables

**Table 1:** Count and classification of lesions in the dogs according to the Fortaleza classification.

Animal	I	II	III	IV	V
D1	0	1	4	0	0
D2	0	4	5	1	0
D3	0	0	99	10	19
D4	0	4	14	1	3
D5	7	4	22	1	0
D6	0	10	8	4	3
D7	0	2	73	2	10
D8	18	1	179	1	0
D9	0	16	5	2	5

**Table 2:** Home range of animals included in the study.

Dogs	Area (m^2^)	CI (95%)
D1	9127.1	8214.2–10,075.7
D2	3767.3	3488.5–4056.5
D3	6502.0	5958.2–7066.6
D4	2310.9	2148.3–2479.8
D5	24,603.2	22,156.7–27,172.6
D6	3956.3	3637.2–4288.5
D7	3817.9	3492.8–4155.6
D8	9478.9	8726.8–10,250.5
D9	38,807.7	32,574.1–45,553.8
Total mean	6502.0	5958.2–7066.6

**Table 3:** Number of samples in each type of area and prevalence within each dog’s home range.

Animal	Peridomicile	Free Area	River	Beach	Positives/Total	Prevalence (%)	CI (95%)
D1	8	4	0	0	1/12	8.33	0–23.97
D2	3	1	0	0	0/4	0	0
D3	1	1	3	0	0/5	0	0
D4	3	0	0	0	0/3	0	0
D5	2	3	10	0	1/15	6.67	0–19.29%
D6	0	0	0	0	0/0	0	0
D7	1	4	4	0	0/0	0	0
D8 *	1	0	0	4	1/5	20.0	0–55.06%
D9 *	0	0	1	0	½	50.0	0–100%

* Animals shared a positive sand sample in their home range.

**Table 4:** Number and percentage of positive and negative samples in relation to the analyzed variables in each collection area.

		Positive		Negative			Fisher Test
Variable	Category	N	%	N	%	Total	
Organic material	Dry	3	1	305	99	308	
	Food	0	0	11	100	11	nr
	Feces	0	0	5	100	5	
	None	0	0	4	100	4	
Animal	Chicken	0	0	57	100	57	
	Dog	3	4.3	67	95.7	70	
	Cat	0	0	2	100	2	nr
	Duck	0	0	1	100	1	
	None	0	0	198	100	198	
Shadow	Morning	0	0	95	100	95	
	Afternoon	0	0	2	100	2	
	Both periods	3	1.6	183	98.4	186	nr
	Absent	0	0	45	100	45	
Soil type	Sand	3	1	299	99	302	F = 1
	Clay soil	0	0	26	100	26	*p* = 1

nr: Analysis not performed due to the number of zeros.

## Data Availability

Data are available within the article.
